# Propofol-Induced Changes in Neurotrophic Signaling in the Developing Nervous System *In Vivo*

**DOI:** 10.1371/journal.pone.0034396

**Published:** 2012-04-04

**Authors:** Jelena Popic, Vesna Pesic, Desanka Milanovic, Smilja Todorovic, Selma Kanazir, Vesna Jevtovic-Todorovic, Sabera Ruzdijic

**Affiliations:** 1 Department of Neurobiology, Institute for Biological Research, University of Belgrade, Belgrade, Serbia; 2 Department of Anesthesiology, University of Virginia Health System, Charlottesville, Virginia, United States of America; Universidade Federal do Rio de Janeiro, Brazil

## Abstract

Several studies have revealed a role for neurotrophins in anesthesia-induced neurotoxicity in the developing brain. In this study we monitored the spatial and temporal expression of neurotrophic signaling molecules in the brain of 14-day-old (PND14) Wistar rats after the application of a single propofol dose (25 mg/kg i.p). The structures of interest were the cortex and thalamus as the primary areas of anesthetic actions. Changes of the protein levels of the brain-derived neurotrophic factor (BDNF) and nerve growth factor (NGF), their activated receptors tropomyosin-related kinase (TrkA and TrkB) and downstream kinases Akt and the extracellular signal regulated kinase (ERK) were assessed by Western immunoblot analysis at different time points during the first 24 h after the treatment, as well as the expression of cleaved caspase-3 fragment. Fluoro-Jade B staining was used to follow the appearance of degenerating neurons. The obtained results show that the treatment caused marked alterations in levels of the examined neurotrophins, their receptors and downstream effector kinases. However, these changes were not associated with increased neurodegeneration in either the cortex or the thalamus. These results indicate that in the brain of PND14 rats, the interaction between Akt/ERK signaling might be one of important part of endogenous defense mechanisms, which the developing brain utilizes to protect itself from potential anesthesia-induced damage. Elucidation of the underlying molecular mechanisms will improve our understanding of the age-dependent component of anesthesia-induced neurotoxicity.

## Introduction

General anesthetics are routinely used in the clinic and their safety is usually determined by the clinical outcome [1]. Propofol (2,6-diisopropylphenol) has widespread use as an agent for the induction and maintenance of anesthesia because of the rapid onset of its essentially short-acting anesthetic effects and minimal side effects. However, the molecular mechanisms that underlie the effects of propofol on neuronal activity remain elusive. Proposed mechanism of action of propofol in the inhibition of neuronal activity is based mainly on the activation of gamma-aminobutyric acid A (GABA_A_) receptors [2]. Since GABA-mediated neuronal activity is essential for brain development, it is plausible that exposure to general anesthetics interferes with normal maturation and continual behavioral deficits of the brain [3], [4].

The neurotrophins are a family of secreted proteins that mediate numerous functions in both the developing and mature nervous system, including growth, survival, differentiation and synaptic plasticity of postmitotic neurons [5]. They are comprised of the NGF, BDNF, neurotrophin 3 (NT-3) and neurotrophin 4/5 (NT4/5) [6]. Neurotrophins bind the Trk receptors, the members of a large tyrosine kinase receptor family [6]. BDNF and NGF exhibit high affinity binding to TrkB and TrkA, respectively [7]. The binding of neurotrophins to Trk receptors induces their dimerization which is followed by autophosphorylation of tyrosine residues within the intracellular kinase domain, that leads to the activation of signaling pathways such as the phosphatidylinositol 3-kinase (PI3K)/Akt and mitogen-activated protein kinase (MAPK)/ERK pathways [5], [8]. Akt and ERK kinases play a crucial role in regulating various processes in the brain, including neuronal proliferation, differentiation, development, migration, survival and long-term synaptic plasticity [9], [10]. Phosphorylated Akt can protect cells from apoptosis via stimulation of the expression of proteins that favor cell survival and by inhibiting executor caspases [11], [12]. Activation of ERK1/2 generally promotes cell survival, although under certain conditions, ERK1/2 can possess pro-apoptotic properties [13]. In our previous study [14], we reported that short-term propofol anesthesia could have a neurotoxic effect in the cortex and thalamus of PND7 rats and that this effect is mediated, at least in part, by neurotrophic downregulation.

In small rodents such as mice and rats, the brain is underdeveloped at birth, but rapidly matures during the first weeks of life in a process known as the brain growth spurt [15]. However, the precise timing of regional brain development and the exact peak of synaptogenesis for each brain region has not been established. It was shown that rat pups are vulnerable to anesthesia early in synaptogenesis, that they reach peak vulnerability around PND7, and that they are much less sensitive at later stages of synaptogenesis [14], [16]. In contrast to PND7 which are particularly sensitive to the neurotoxic effects of anesthetics, postnatal day 14 (PND14) being a less vulnerable stage anesthesia-wise. It is therefore assumed that anesthetics do not exert neurotoxic effects at this developmental stage [16]. Considering that short-term propofol anesthesia is widely used in pediatric practice in all age groups, it is essential to experimentally determine whether the neurotoxic effects of a single propofol dose are age-dependent and whether neurotrophic imbalance contributes to this process.

To this end, we wanted to test the hypothesis whether the upregulation of pro-survival Akt and ERK kinases was sufficient to rescue cells from developmentally-regulated anesthesia-induced neurotoxicity. We expect that the elucidation of Akt/ERK molecular interaction will help unravel the mechanisms that mediate propofol-induced signaling in the brain in the later phase of synaptogenesis. Our results revealed that propofol-induced modulation of neurotrophins in a region-specific manner initiated substantial changes in downstream pro-survival kinases, thereby rescuing cells from neurodegeneration during a critical period of development.

## Results

### Propofol treatment differentially affects protein levels of BDNF in the cortex and thalamus of PND14 rats

**Figure 1:**
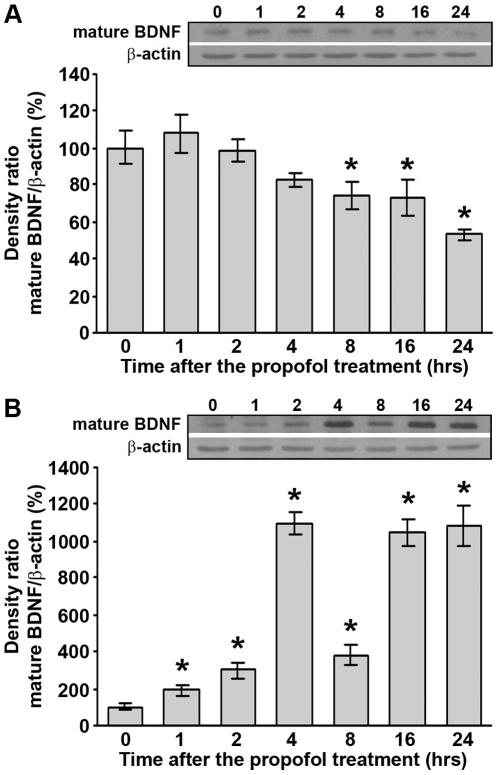
Propofol treatment differentially affects mature BDNF protein levels in PND14 rats. Western blot analysis was used to determine the expression of mature BDNF in the cortex (A) and thalamus (B). Graphs that show changes in protein levels are accompanied by representative immunoblots. The data are expressed as percentages relative to the respective controls (mean ± SEM): *p<0.05 vs. control.

To investigate whether propofol treatment affects neurotrophin signaling, we first monitored the changes in mature BDNF protein levels in two brain areas involved in anesthetic actions: the cortex and the thalamus. Western blot analysis revealed a significant downregulation of mature BDNF protein (14 kDa) in the cortex (Fig. 1A, *p<0.05), and significant upregulation in the thalamus after the propofol treatment (Fig. 1B, *p<0.05). A significant decrease of mature BDNF protein in the cortex was detected at 8 h post-treatment. This trend was sustained until 24 h post-treatment when a maximal decline was recorded (50%).

In the thalamus, a significant increase was detected at 1 h post-treatment (2-fold). Moreover, pronounced upregulation of BDNF levels (more than 10-fold) at time points 4, 16 and 24 h after the treatment was observed. Statistical analyses revealed a significant effect of the time after the treatment for the cortex (one-way ANOVA: F(6, 28) = 6.497, p = 0.001) and for the thalamus (Kruskal-Wallis ANOVA: H(6, N = 56) = 46.671, p = 0.001).

### Propofol treatment differentially affects protein levels of total and phosphorylated TrkB in the cortex and thalamus of PND14 rats

**Figure 2:**
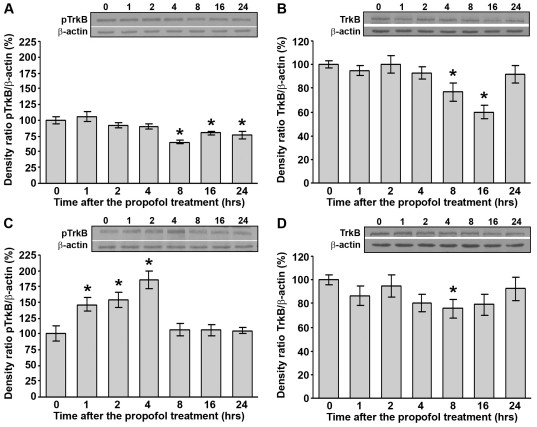
Propofol treatment differentially affects protein levels of total and phosphorylated TrkB in PND14 rats. Western blot analysis was used to determine the expression of pTrkB and TrkB receptors in the cortex (A and B, respectively) and thalamus (C and D, respectively). Each graph is accompanied by a representative immunoblot. The data are expressed as percentages relative to the respective controls (mean ± SEM): *p<0.05 vs. control.

Since BDNF expression was affected by the propofol treatment, we next examined whether these changes influence the expression of its respective receptor TrkB. In the cortex, Western blot analysis did not reveal any significant changes in pTrkB (140 kDa) expression until 8 h post-treatment when a significant decrease (by 20–40%) was observed which lasted until the end of follow-up period (Fig. 2A, *p<0.05). In contrast with the findings obtained in the cortex, the level of pTrkB protein in the thalamus was upregulated during the 1–4 h post-treatment period (50–80%) (Fig. 2C, *p<0.05). However, during the 8–24 h post-treatment period, the levels of pTrkB were stable, remaining at the control level. Statistical analyses (one-way ANOVA) revealed a significant effect of the time after the treatment for both structures (cortex: F(6, 28) = 8.321, p = 0.001; thalamus: F(6, 35) = 9.474, p = 0.001).

In addition, we monitored the expression of total, unphosphorylated TrkB receptor (140 kDa). Unphosphorylated TrkB receptor was downregulated from 8 to 16 h post-treatment in the cortex (25–40%) (Fig. 2B, *p<0.05), and only at 8 h post-treatment in the thalamus (25%) (Fig. 2D, *p<0.05). Statistical analyses (one-way ANOVA) revealed a significant effect of the time after the treatment only in the cortex (F(6, 49) = 6.524, p = 0.001). In the thalamus, post hoc analyses by the LSD test revealed a significant effect of the time at 8 h after treatment (*p<0.05).

### Propofol treatment down-regulates NGF protein levels in the cortex and thalamus of PND14 rats

**Figure 3:**
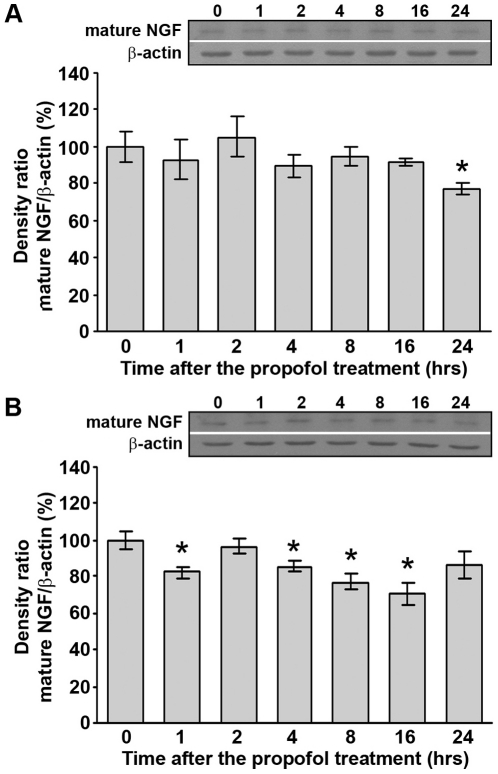
Propofol treatment down-regulates mature NGF protein levels in PND14 rats. Western blot analysis was used to determine the expression of mature NGF in the cortex (A) and thalamus (B). Graphs that show changes in protein levels are accompanied by representative immunoblots. The data are expressed as percentages relative to the respective controls (mean ± SEM): *p<0.05 vs. control.

We also wanted to investigate whether propofol treatment affects the expression of other neurotrophins such as NGF. Western blot analysis showed that propofol treatment did not affect the expression of mature NGF protein (14 kDa) in the cortex except at 24 h post-treatment when a slight but significant decrease (by about 20%) was detected (Fig. 3A, *p<0.05).

In the thalamus, decreased levels (20–30%) of mature NGF protein were detected at all time points except at 2 and 24 h post-treatment (Fig. 3B, *p<0.05). Statistical analyses (one-way ANOVA) revealed a significant effect of the time after the treatment only in the thalamus (F(6, 28) = 4.23, p = 0.004) and in the cortex, *post hoc* analyses by the LSD test revealed a significant effect of the time at 24 h after treatment (*p<0.05).

### Propofol treatment up-regulates phosphorylated TrkA protein levels in the cortex and thalamus of PND14 rats

**Figure 4:**
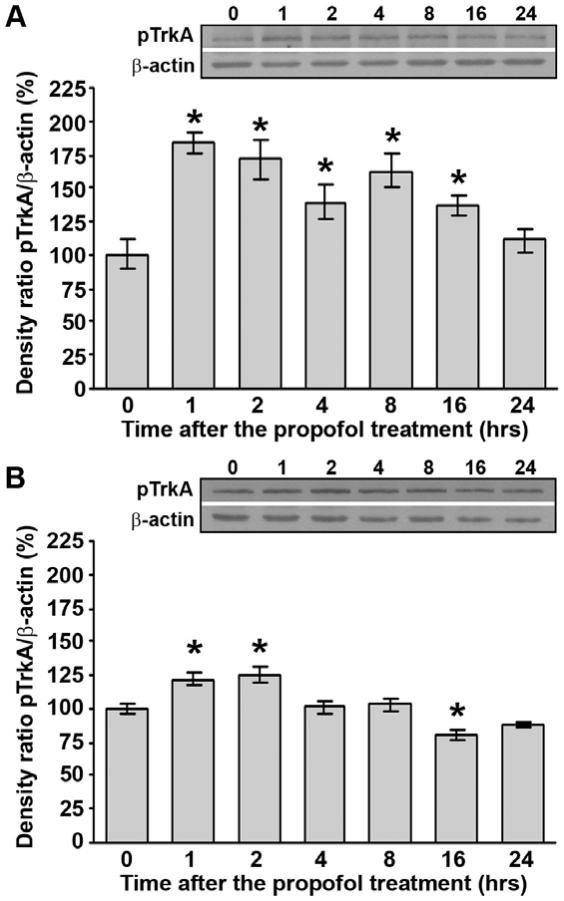
Propofol treatment up-regulates phosphorylated TrkA protein levels in PND14 rats. Western blot analysis was used to determine the expression of pTrkA in the cortex (A) and thalamus (B). Graphs that show changes in protein levels are accompanied by representative immunoblots. The data are expressed as percentages relative to the respective controls (mean ± SEM): *p<0.05 vs. control.

Since NGF expression was affected by propofol treatment, we examined whether this influences the expression of its activated TrkA receptor. The propofol treatment led to an increase (by 40–85%) of pTrkA (140 kDa) expression in the cortex (Fig. 4A, *p<0.05) at 1 to 16 h post-treatment.

In the thalamus, propofol treatment led to a slight increase (by about 25%) in the level of pTrkA at 1 and 2 h post-treatment (Fig. 4B, *p<0.05). At 4 to 24 h, the expression of pTrkA was the same as in the control, albeit a transient decrease was observed at 16 h post-treatment (Fig. 4B, *p<0.05). Statistical analyses (one-way ANOVA) revealed a significant effect of the time after the treatment for both structures (cortex: F(6, 42) = 7.939, p = 0.001; thalamus: F(6, 35) = 14.956, p = 0.001).

### Propofol treatment differentially affects protein levels of total and phosphorylated Akt in the cortex and thalamus of PND14 rats

**Figure 5:**
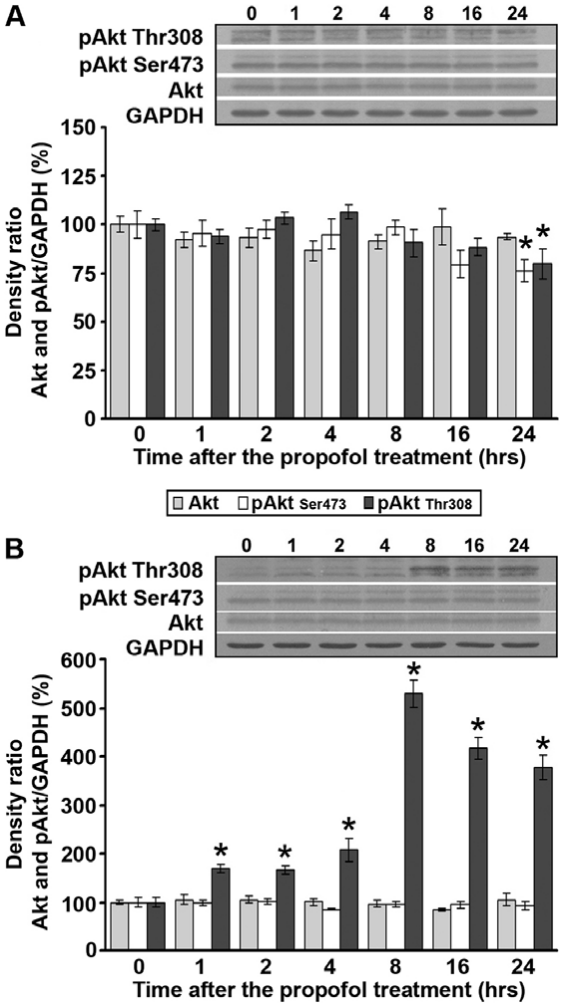
Propofol treatment differentially affects protein levels of total and phosphorylated Akt in PND14 rats. Western blot analysis was used to determine the expression of Akt and pAkt (Thr308 and Ser473) kinase in the cortex (A) and thalamus (B). Each graph is accompanied by representative immunoblots. The data are expressed as percentages relative to the respective control (mean ± SEM): *p<0.05 vs. control.

We next examined by Western blot analysis whether activation of neurotrophic receptors can influence temporal patterns of total Akt and activated (phosphorylated) pAkt protein expression. The propofol treatment did not change the levels of total Akt (56/60 kDa) in the cortex (Fig. 5A) nor did it affect pAkt (Thr308 and Ser473) up to 24 h post-treatment. At this time point, decreased levels of both pAkt (Thr308) and pAkt (Ser473) (by about 25%) were detected (Fig. 5A, *p<0.05). Regardless of the fact that the LSD test revealed the significance, one-way ANOVA did not reveal a significant effect of time after the treatment.

As in the cortex, the propofol treatment did not change the levels of total Akt protein in the thalamus (Fig. 5B). The levels of pAkt (Ser473) were unaffected by the treatment as well. In contrast, a significant increase of pAkt (Thr308) was detected at all time points after the treatment, with the most dramatic increase (4–5-fold) observed in the thalamus during the 16–24 h period after the treatment. Kruskal-Wallis ANOVA revealed a significant effect of the time after the treatment (H(6, N = 21) = 18.944, p = 0.004).

### Propofol treatment differentially affects protein levels of total and phosphorylated ERK1/2 in the cortex and thalamus of PND14 rats

**Figure 6:**
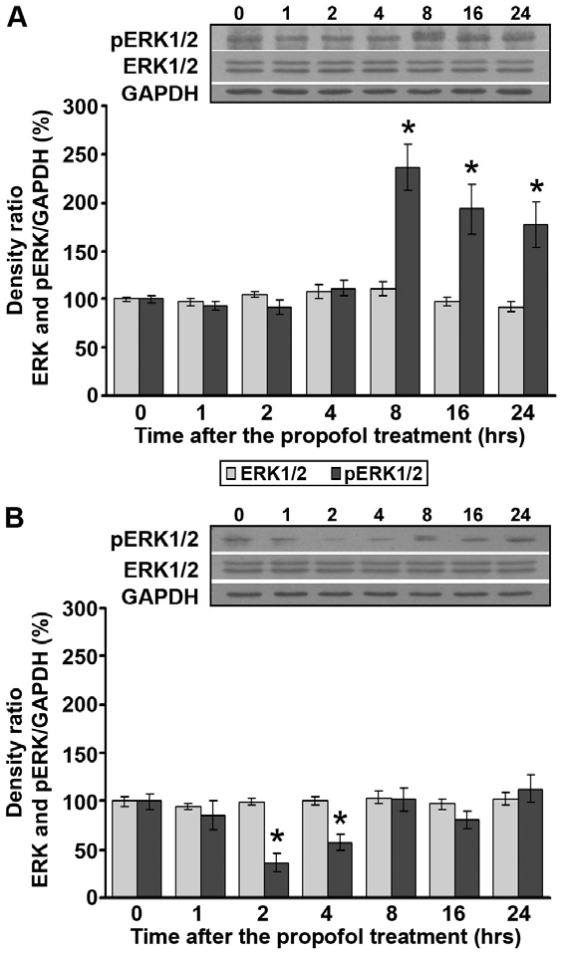
Propofol treatment differentially affects protein levels of total and phosphorylated ERK1/2 in PND14 rats. Western blot analysis was used to determine the expression of ERK1/2 and pERK1/2 kinase in the cortex (A) and in the thalamus (B). Each graph is accompanied by representative immunoblots. The data are expressed as percentages relative to the respective control (mean ± SEM): *p<0.05 vs. control.

We evaluated by Western blot analysis the levels of total and phosphorylated ERK1/2 protein (42/44 kDa), one of the main downstream effector kinases in the neurotrophic signaling pathway. In the cortex, the propofol treatment did not change the expression of total ERK1/2 protein (Fig. 6A). In contrast, an increased level of pERK1/2 (2–2.5-fold) was detected in the same structure during the 8–24 h post-treatment period (Fig. 6A, *p<0.05) (Kruskal-Wallis ANOVA: H(6, N = 35) = 26.149, p = 0.002).

As in the cortex, the propofol treatment did not change the expression of total ERK1/2 in the thalamus (Fig. 6B) whereas it drastically decreased (by 40–60%) the expression of pERK1/2 during the 2–4 h post-treatment period (Fig. 6B, *p<0.05). Thereafter, phosphorylation of ERK1/2 returned to the basal level (One-way ANOVA: F(6, 21) = 5.732, p = 0.001).

### Propofol treatment did not induce neurodegeneration despite the presence of the caspase-3 active fragment in the cortex and thalamus of PND14 rats

**Figure 7:**
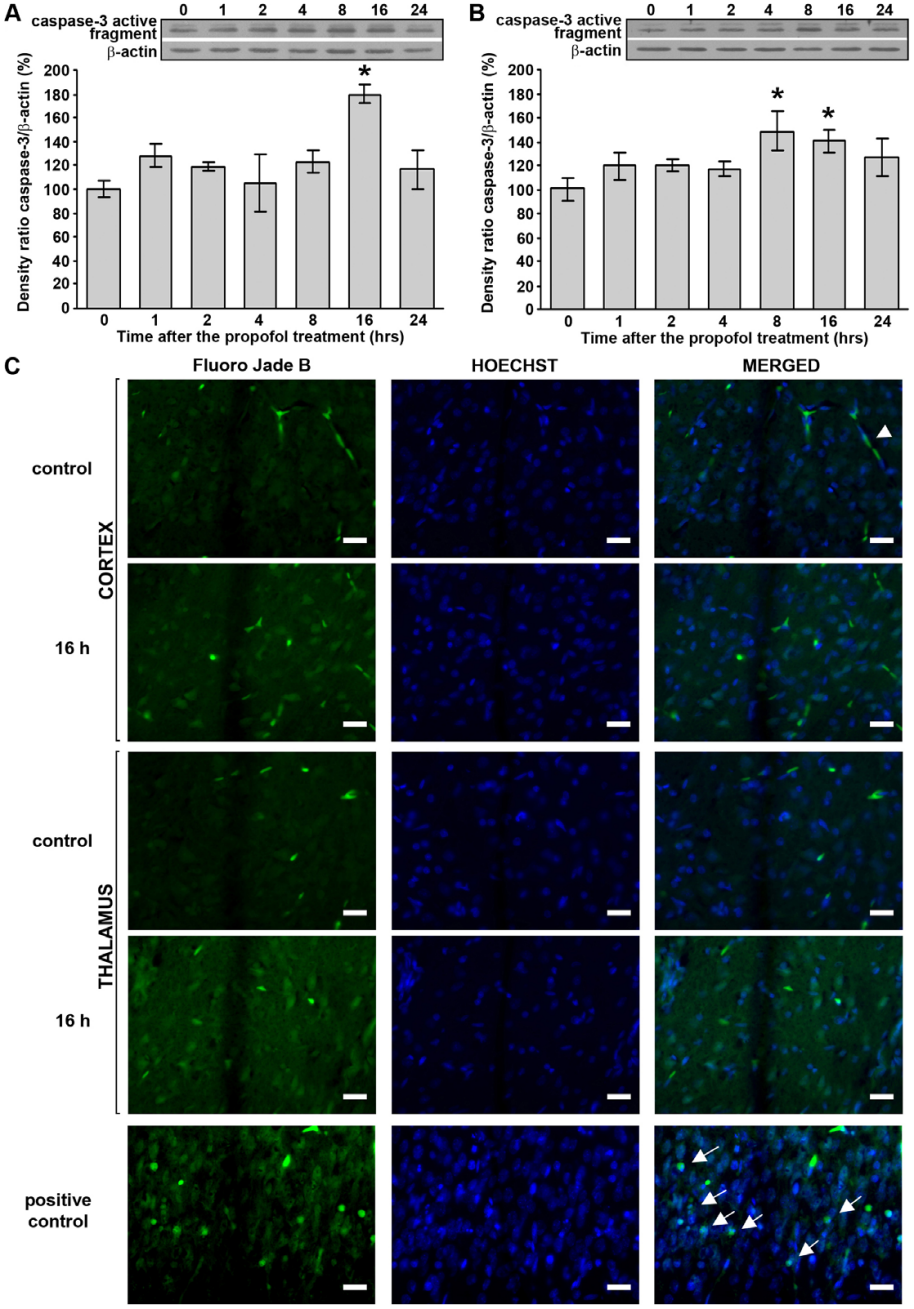
Propofol treatment did not induce neurodegeneration even with detected caspase-3 active fragment in PND14 rats. Expression of the caspase-3 active fragment in the cortex (A) and thalamus (B). The data are expressed as percentages relative to the respective control (mean ± SEM), *p<0.05 vs. control. (C) Fluoro-Jade B staining (left panel), Hoechst 33258 staining (middle panel) and merged images (right panel) of representative brain sections of the cortex and the thalamus from control-(1^st^ and 3^rd^ row, respectively) and propofol-treated (16 h) (2^nd^ and 4^th^ row, respectively) PND14 animals. PND7 rats that served as a positive controls were treated with two i.p. doses (0.5 mg/kg) of (+) MK-801 and killed 24 h after the first administration of the drug. Degenerating neurons are marked with arrows and blood vessels with arrowheads. Scale bar = 20 µm.

To determine whether the changes in neurotrophin signaling that were induced by the propofol treatment influence cell survival in our experimental model, we examined the expression of cleaved caspase-3, which served as a marker of apoptosis. Western blot analysis showed that in the cortex, the propofol treatment induced the expression of caspase-3 active fragment (17/19 kDa) at 16 h post-treatment (Fig. 7A). In the thalamus, propofol treatment stimulated the expression of caspase-3 active fragment (by about 50%) at 8 h after the treatment; this effect was maintained up to the 16 h time point after which it returned to the control level (Fig. 7B). Statistical analyses (one-way ANOVA) revealed a significant effect of the time after the treatment only in the cortex (F(6, 21) = 4.217, p = 0.006). In the thalamus, *post hoc* analyses by the LSD test revealed a significant effect of the time at 8 and 16 h after treatment (*p<0.05).

Since Western blot analysis revealed an increase in caspase-3 active fragment in both examined structures at 16 h post-treatment, we investigated whether neurodegenerative changes are detectable at the histological level at 8, 16 and 24 h after the propofol treatment. To this end, we stained sections with Fluoro-Jade B. Along with Fluoro-Jade B, the sections were labeled with the nucleic acid stain Hoechst 33258. As a positive control, we used PND7 rats that were treated with two i.p. doses (0.5 mg/kg) of (+) MK-801 and killed 24 h after the first administration of the drug (Fig. 7C, last row). Fluoro-Jade B positive cells were not detected in the cortex and the thalamus either in control (Fig. 7C, 1st and 3rd row, respectively) or in brain slices from treated rats (Fig. 7C, 2nd and 4th row, respectively). Furthermore, in order to determine what dose of propofol in PND14 rats promotes cell death, we performed independent Fluoro-Jade B experiments on rats that were separated into five groups: control, treated with the following doses of propofol – 25, 50, 75, 100 mg/kg. The rats were killed 16 h post-treatment. We did not find any degenerating neurons in the cortex and thalamus in any of the examined groups, except in the group treated with a 100 mg/kg dose of propofol where we observed only one degenerating cell per examined structures (data not shown).

## Discussion

Following administration of a single dose of propofol, we examined the temporal and spatial expression of neurotrophins BDNF and NGF, their activated receptors (TrkB and TrkA, respectively) and downstream kinases (Akt and ERK) in the cortex and in the thalamus of PND14 rats, two brain areas involved in the actions of anesthetics. The obtained results show that marked changes in the levels of the examined neurotrophins, their receptors and downstream effector kinases that were induced by the treatment, were not associated with increased cell death in the cortex and thalamus.

In this study, the changes in mature BDNF protein expression were region-specific and dynamically modulated during the first 24 h after a single propofol treatment. We observed a robust propofol-induced increase in mature BDNF protein in the thalamus of PND14 rats. This contrasts the results obtained in PND7 rats, in which thalamic levels of BDNF were reduced by same propofol treatment (Pesic et al., paper in preparation) or by anesthesia cocktail containing midazolam, isoflurane and nitrous oxide [17]. Increase in the mature BDNF protein in the thalamus of PND14, but not PND7 rats could also be explained by the retrograde transport of mature BDNF [18]. BDNF can also be transported anterogradely in the CNS and therefore acts as both a target-derived neurotrophic factor and an autocrine/paracrine modulator [19]. In contrast to the thalamus, the levels of mature BDNF protein appear to be downregulated in the cortex. This is in agreement with other rodents studies where propofol treatment led to downregulation of BDNF in the parietal cortex [20]. BDNF transcription and secretion is known to depend on the release of Ca^2+^ from intracellular depots [21]–[23]. Since propofol influences Ca^2+^ homeostasis [24] it is thus plausible that the differences in the expression of BDNF protein in the thalamus and the cortex are due to their differential sensitivity to the propofol-induced Ca^2+^ flux. Altogether, our result could point to fast BDNF turnover related to redistribution to, and from, different brain regions in the case of the cortex and thalamus, respectively. We can assume that propofol treatment rapidly regulates BDNF at posttranslational level, but this process needs to be further investigated.

The propofol-induced decrease in the levels of mature BDNF in the cortex (observed 8–24 h period after the treatment) was accompanied by decreased expression of phosphorylated TrkB receptor. On the other hand, the increased expression of mature BDNF in the thalamus (at all experimental time points), was accompanied by increased expression of pTrkB only during the 1–4 h period after the treatment but not at other times. In addition, *in vitro* experiments employing compartmentalized cultures have shown that pTrk receptors accumulate in cell bodies and in proximal axons of sympathetic and sensory neurons after exposure to neurotrophins [25]–[27]. Therefore, the results obtained in our study suggest that in PND14 brains, the alterations in BDNF expression induced by propofol treatment were probably extracellular. It is for this reason that the appropriate modulation in TrkB phosphorylation (activity) could be detected. We also examined the treatment-induced changes in total, unphosphorylated TrkB receptor. We observed the downregulation of total TrkB receptor during the 8–16 h period after treatment, while in the thalamus decreased expression of the receptor was detected only at 8 h post-treatment. Thus, in the light of the results obtained for BDNF and TrkB receptors (both total and phosphorylated), it can be hypothesized that the slight decrease in BDNF level mostly triggers rapid changes in receptor number, while a robust increase in BDNF level is mostly related to modifications in receptor activity. Moreover, we think that the results obtained in the thalamus clearly show how a system defends itself from excessive ligand levels and prolonged receptor activation. This was expected considering the important roles that BDNF assumes during early postnatal development.

As for the other investigated neurotrophin, we detected downregulation of mature NGF in both examined brain structures. While the levels of mature NGF decreased, TrkA receptor was hyperphosphorylated in both the cortex and the thalamus. Thus, in our experimental model, the changes in NGF expression did not appear to alter the activity of the TrkA receptor as was shown previously [28]. However, *in vitro* studies have shown that in addition to NGF, other ligands can phosphorylate TrkA receptor, and that auto-activation of TrkA in the absence of ligands is possible as well [8], [29]. Therefore, it would appear that propofol-induced changes in NGF and pTrkA in the cortex and thalamus of PND14 rats could be explained, but comprehensive molecular analysis is necessary to elucidate the details of these events.

Akt is a well-known cellular pro-survival kinase that acts downstream from neurotrophin receptors [30], [31]. In response to growth factors, Akt is phosphorylated at two major sites: Thr308 and Ser473 [31]–[33]. We observed that after propofol treatment, pAkt Thr308 is downregulated in the cortex and upregulated in the thalamus. This roughly parallels the propofol-induced changes in mature BDNF and pTrkB receptor. Surprisingly, changes in pAkt Ser473 were observed only in the cortex, with downregulation at the same time point when the level of pAkt Thr308 was decreased. Together with the finding that the treatment did not induce changes in total Akt level in both examined structure, our results imply that the propofol-induced changes were related to modified kinase activity. While only two studies investigated changes in pAkt level after the administration of an anesthetic to the developing rats [14], [17], they did not examine the phosphorylation of both sites. It remains to be investigated why in the cortex decreased phosphorylation of Thr308 was followed by decreased phosphorylation of Ser473, while in the thalamus increased phosphorylation of Thr308 was not followed by the same modifications on Ser473. Since Akt is one of the major regulators of many important cellular functions, it is possible that partial Akt phosphorylation serves to protect the system from hyperphosphorylated and over-activated Akt kinase, which could ultimately have deleterious consequences [34].

In addition to Akt, ERK critically influences cell survival and acts as an effector of neurotrophin signaling [35]. The influence of propofol on ERK phosphorylation has been mainly examined in *in vitro* studies and the obtained results are contradictory [36]–[38]. Our study showed that pERK1/2 is upregulated in the cortex and downregulated in the thalamus of PND14 rats upon propofol administration in a time-dependent manner. Increased pERK in the cortex could result from propofol-induced activation of pTrkA receptor. It has been suggested that TrkA internalization is a key event for sustained ERK signaling in PC12 cells [39], and that sustained activation of MAPK activity is a distinguishing feature of neurotrophin signaling [40]. As for the thalamus, we propose that the observed changes in pERK1/2 are the consequence of propofol-induced modulation of Akt signaling. It has been demonstrated that Akt inhibits ERK [41]–[43]. To date, reciprocal regulation of Akt and ERK proteins by propofol has not been observed *in vivo*.

Several studies in which anesthesia were administered at earlier developmental stages (P0–P10) have reported that general anesthetics downregulate the expression of neurotrophins, leading to subsequent neuroapoptosis [14], [17]. In contrast, we monitored the effects of propofol treatment in PND14 animals when GABA depolarizing-to-hyperpolarizing shift has already occurred [44], [45]. While the active caspase-3 fragment was detected in both examined structures, we did not observe neurodegeneration at the histological level. This is most likely due to the effects of propofol treatment on Akt and ERK1/2 in the thalamus and cortex, respectively. In turn, these pathways could stimulate anti-apoptotic proteins such as X-linked inhibitor of apoptosis protein (XIAP) which binds and inhibits activated caspase-3 [46], as was recently demonstrated in PC12 cells [47]. Although caspase-3 is often used as a marker of apoptosis, the activation of apoptotic proteases can occur transiently and in the absence of cell death. Apoptotic biochemical cascades can exert local actions and serve physiological roles such as sculpting neuritic architecture and synaptic connectivity in developing and in mature nervous system [48]. In addition, it has recently been shown that propofol can attenuate the isoflurane-induced caspase-3 activation *in vivo* and *in vitro* and thus act as neuroprotective agent [49]. Thus, our results are in agreement with other studies in which general anesthetics did not exhibit neurotoxic properties [50], [51]. Together with the aforementioned studies, our results suggest that the neurotoxic effects of general anesthetics largely depend on the developmental stage of the animal.

In conclusion, this is the first study in which propofol has been described as an agent that exerts complex effects on neurotrophins and their downstream signaling pathways in the developing brain. Our data suggest that the expression of BDNF and NGF is differentially regulated in response to the propofol treatment and that the main changes in PND14 rat brain occur via the BDNF signaling pathway and to a lesser extent via the NGF signaling pathway. We believe that the overexpression of mature BDNF observed in our experimental model is not exclusively limited to neuronal survival, but can also affect other aspects of neural plasticity (paper in preparation). In addition to the significant changes in expression of neurotrophins, the treatment induced changes in Akt and ERK expression in a region-specific manner. Fluoro-Jade B histochemistry did not reveal neuronal degeneration, despite the observed transient increase in the expression of the caspase-3 active fragment. The obtained results indicate that in the brain of PND14 rats the activation of Akt and ERK signaling are an important aspect of the endogenous defense mechanisms that protect the developing brain from potential damage. The balanced expression of these pro-survival kinases in the same brain region seems to be important for proper output signaling and a favorable physiological outcome. In light of the previous results [14], it can be assumed that anesthesia-induced neurotoxicity in the developing brain is determined by age-dependent peculiarities in the balance between competing pro- and anti-apoptotic signals. Together, our results contribute towards an improved understanding of the actions of anesthetics in the developing brain *in vivo*; they also bare potential clinical implications.

## Materials and Methods

### Ethics Statement

All experiments were approved by the institutional committee and the University of Virginia Animal Care and Use Committee, and were performed in accordance with the Guide for the Care and Use of Laboratory Animals (National Institute for Health, Publication No. 85/23).

### Animals and Treatment

Fourteen-day-old (PND14) male Wistar rats (average body weight 40–42 g) were used in all experiments. Animals were treated with propofol manufactured for i.v. human use (Recofol®). The formulation contained in addition to the active substance, soybean oil, purified egg phosphatide, glycerol and sodium hydroxide to adjust the pH. The ampoules were shaken well and the drug was used according to the manufacturer's instructions.

The procedures were designed to minimize the suffering of the animals and the number of rats used. Rat pups were placed in a temperature-controlled incubator set to an ambient temperature of 35–36°C. Animals not intended to be killed immediately after anesthesia were allowed to recover in the incubator for 1 h and were returned to their mothers to feed. Loss of the righting reflex (LRR) served as an indicator of anesthetic-induced unconsciousness and sleeping time. The length of time required for LRR was defined as the time between the injection and the point at which the animal could no longer successfully perform the righting response when placed on its back. The times of loss and recovery of the righting reflex, measured in minutes, were recorded with a stopwatch. We also used the tail pinching test to examine pain sensation in the anesthetized animals.

We first performed an independent experiment in order to obtain the optimal dose of propofol that was capable of inducing effective anesthesia and which would be used in the main study. PND14 rats were intraperitoneally injected with 10, 25, 50, 75 and 100 mg/kg of propofol. Animals that received the lowest dose of propofol (10 mg/kg, i.p.) were sluggish but remained fully awake. This was the only dose that had no effect on the righting reflex. All other doses caused loss of the righting reflex within the first couple of minutes; this was accompanied by the absence of response to pain stimuli (tail pinch test). The righting reflex was impaired for about 20, 45, 110, 170 min in rats that were administered 25, 50, 75 and 100 mg/kg doses of propofol, respectively. Propofol doses of 25 and 50 mg/kg did not cause changes in skin color and breathing patterns. No animal died during these experiments. Since the injecting volume was too high for animals that received propofol at a dose of 100 mg/kg, we did not examine higher doses. Based on these observations and on our previous studies on PND7 animals, we performed all subsequent experiments with a propofol dose of 25 mg/kg, which impaired the righting reflex for 20±2 min.

### Experimental Procedure

PND14 rat pups (n = 40) were separated from their mothers and placed in a temperature-controlled incubator set to an ambient temperature of 35°C. The animals were randomly divided into seven groups (n = 4 per group) and were administered a bolus injection of propofol (25 mg/kg, i.p.). The animals were decapitated at different time points after the treatment (at 0, 1, 2, 4, 8, 16 and 24 h). Animals that were not killed immediately after the anesthesia were allowed to recover in the incubator for 1 h and were returned to their mothers to feed. The cortex and the thalamus from both hemispheres were isolated and frozen. Since small amounts of tissue were isolated *per* animal, the samples were pooled (n = 4 per group).

For Fluoro-Jade B staining, the animals were divided into four groups (n = 3 per group). Three groups were propofol injected (25 mg/kg i.p.) and decapitated 8, 16 and 24 h after the treatment. The last group, the physiological control, was injected an appropriate volume of saline. Whole brains were isolated from these animals.

Ventilation, oxygenation and perfusion in pups were not measured due to technical difficulties; however, the animals were closely monitored throughout the experiment. All pups appeared pink, well perfused and with no visible signs of cyanosis. We did not examine the effect of the vehicle (lipid emulsion) because the aim of the experiments was to explore the effects of propofol in the form used clinically on humans, also, it was shown previously that the lipid vehicle does not have any effect [52]–[55].

### Tissue Preparation

To obtain whole-cell extracts, the tissue was homogenized in 10 volumes of RIPA buffer (50 mM Tris–HCl pH 7.5, 150 mM NaCl, 1% NP-40, 0.1% SDS, 0.5% Triton X-100, 1 mM EDTA pH 8.0, 1 mM EGTA pH 7.2) containing protease and phosphatase inhibitors. The homogenates were sonicated and centrifuged at 14 000 g for 30 min at 4°C. The supernatants were collected and stored at −80°C until use.

Protein concentrations were determined by the bicinchonic acid micro-protein assay (Micro BCA Protein Assay Kit; Pierce Inc., Rockford, USA) with albumin as standard.

### Western Blot Analysis

Twenty micrograms of protein extracts were heat denaturized, separated by 12% SDS polyacrylamide gel electrophoresis and transferred to nitrocellulose membranes (Amersham Bioscience, Otelfingen, Switzerland). The membranes were blocked at room temperature for 1 h either in 3% bovine serum albumin or in 5% nonfat dry milk in Tris-buffered saline/0.1% Tween-20 (TBS-T), followed by incubation for 2 h at room temperature or overnight at 4°C with primary antibodies diluted in blocking buffer. The membranes were incubated for 1 h at room temperature with secondary antibodies in Tris-buffered saline/0.1% Tween-20. Three washes with 0.3% Tween-20 in Tris-buffered saline were performed between all steps. All blots were incubated with anti-β-actin or anti-GAPDH antibodies to correct for any differences in protein loading.

Immunoreactivity was detected by enhanced chemiluminescence (ECL; Amersham Bioscience) after exposure on an X-ray film. All films were densitometrically analyzed using the computerized image analysis program ImageQuant 5.0. The individual samples that were examined were pooled from all of the animals that belonged to the same group (n = 4 per group). Therefore, for each analyzed protein, five different blots were densitometrically analyzed. Each blot contained a set of seven samples that were matched according to the time of decapitation (0, 1, 2, 4, 8, 16, 24 h after the treatment).

The following antibodies were used in this study: rabbit polyclonal BDNF (N-20: sc-546), rabbit polyclonal NGF (H-20: sc-548), rabbit polyclonal Trk B (sc-12), mouse monoclonal p-Trk A (E-6: sc-8058), rabbit polyclonal Akt1/2/3 (H-136: sc-8312), rabbit polyclonal p-Akt1/2/3 (Thr-308)-R (sc-16646-R), rabbit polyclonal p-Akt1/2/3 (Ser-473)-R (sc-7985-R), rabbit polyclonal ERK 1 (K-23: sc-94), mouse polyclonal p-ERK (E-4: sc-7383), goat polyclonal GAPDH (sc-20357), bovine anti-goat IgG-HRP (sc-2350), bovine anti-rabbit IgG-HRP (sc-2370). All were purchased from Santa Cruz Biotechnology, Inc., Santa Cruz, USA. Rabbit polyclonal TrkB (phospho Y816) (ab75173) was from Abcam, Cambridge, USA; rabbit polyclonal cleaved caspase-3 (Asp175, #9661) was from Cell Signaling Technology, Beverly, USA; rabbit polyclonal β-actin (A-5060) was from Sigma, Saint Louis, USA; and rabbit polyclonal anti-mouse IgG-HRP (P0260) was from DACO, Glostrup, Denmark.

### Fluoro-Jade B and Hoechst 33258 staining

The animals (n = 3 per group) were killed 0, 8, 16 and 24 h after the propofol injection. The brains were removed and fixed overnight at 4°C in 4% paraformaldehyde in phosphate-buffered saline (PBS). The brains were cryoprotected by three 24 h incubations at 4°C in solutions of increasing sucrose concentration (10%, 20% and 30%) in PBS. The brains were frozen in isopentane and stored at −80°C. Every fifth coronal section (18 µm thick) was taken, mounted on the slides, and allowed to dry overnight and stored at −20°C. The slides were first immersed in a basic alcohol solution consisting of 1% NaOH in 80% ethanol, distilled water and incubated in 0.06% KMnO_4_ solution for 10 min. The slides were transferred for 10 min to a 0.0001% solution of Fluoro-Jade B (AG310, Chemicon International, Temecula, USA) dissolved in 0.1% acetic acid. The slides were rinsed by three changes of distilled water for 1 min per change. The slides were than immersed in 0.01% Hoechst 33258 (Acros Organics, Fair Lawn, USA) staining solution for 10 min and coverslipped with glycerol.

The tissue sections were examined using an Axio Observer Microscope Z1 (Zeiss, Jena, Germany) with a filter system suitable for visualizing fluorescein isothiocyanate (FITC). Cells labeled with Fluoro-Jade B were observed as individual shiny green spots that were clearly discernible from the background. The (+) MK-801-treated PND7 rats from our previous study served as a positive control. PND7 rats that served as positive controls were treated with two i.p. doses (0.5 mg/kg) of (+) MK-801 and killed 24 h after the first administration of the drug.

### Statistical analysis

The relative changes in protein levels are presented as percentages (mean ± SEM) of the control samples that were assumed to be 100%. Comparisons between the groups were performed using one-way ANOVA, followed by the Fisher LSD test, or by Kruskal-Wallis analysis that was followed by the U-test for the data sets that did not have a normal distribution (Statistica version 5.0; StatSoft, Tulsa, USA). Significance was set at p<0.05.
